# Characterization of an Innovative Biomaterial Derived From Human Wharton’s Jelly as a New Promising Coating for Tissue Engineering Applications

**DOI:** 10.3389/fbioe.2022.884069

**Published:** 2022-06-13

**Authors:** Adrien Fayon, Deborah Helle, Gregory Francius, Jean-Baptiste Vincourt, Véronique Regnault, Dominique Dumas, Patrick Menu, Reine El Omar

**Affiliations:** ^1^ Université de Lorraine, CNRS, IMoPA, Nancy, France; ^2^ CNRS, Laboratoire de Chimie Physique et Microbiologie pour les Matériaux et l’Environnement, Université de Lorraine, Nancy, France; ^3^ Université de Lorraine, CNRS, INSERM, IBSLor (UMS2008/US40), Nancy, France; ^4^ Université de Lorraine, INSERM, DCAC, Nancy, France

**Keywords:** Wharton’s jelly, human extracellular matrix, biomaterial, coating, tissue engineering

## Abstract

The extracellular matrix (ECM) offers the opportunity to create a biomaterial consisting of a microenvironment with interesting biological and biophysical properties for improving and regulating cell functions. Animal-derived ECM are the most widely used as an alternative to human tissues that are of very limited availability. However, incomplete decellularization of these tissues presents a high risk of immune rejection and disease transmission. In this study, we present an innovative method to extract human ECM derived from the Wharton’s jelly (WJ-ECMaa) of umbilical cords as a novel biomaterial to be used in tissue engineering. WJ-ECMaa was very efficiently decellularized, suggesting its possible use in allogeneic conditions. Characterization of its content allowed the identification of type I collagen as its main component. Various other matrix proteins, playing an important role in cell adhesion and proliferation, were also detected. WJ-ECMaa applied as a surface coating was analyzed by fluorescent labeling and atomic force microscopy. The results revealed a particular arrangement of collagen fibers not previously described in the literature. This biomaterial also presented better cytocompatibility compared to the conventional collagen coating. Moreover, it showed adequate hemocompatibility, allowing its use as a surface with direct contact with blood. Application of WJ-ECMaa as a coating of the luminal surface of umbilical arteries for a use in vascular tissue engineering, has improved significantly the cellularization of this surface by allowing a full and homogeneous cell coverage. Taking these results together, our novel extraction method of human ECM offers a very promising biomaterial with many potential applications in tissue engineering such as the one presented direct in vascular tissue engineering. Further characterization of the composition and functionality will help explore the ways it can be used in tissue engineering applications, especially as a scaffold or a surface coating.

## 1 Introduction

Disease or trauma to the human body can lead to tissue damage and degeneration, requiring repair, replacement or regeneration (Langer and Vacanti, 1993). Common methods of treatment include autologous tissue grafts (mammary arteries or saphenous veins for coronary bypass), donor tissue grafts (kidney transplantation) and the use of biomaterials. Autologous tissue is an ideal biological material, but unfortunately it is only available in limited quantities, the recovery method can be invasive and often it may not be available due to the critical condition of the patient or due to prior use. To provide suitable alternatives for tissue regeneration or defective organ replacement, the field of tissue engineering has emerged (Griffith and Naughton, 2002). Tissue engineering requires an optimal combination of cells, scaffold(s) and a suitable microenvironment (medium, growth factors, mechanical influences, etc.) (Berthiaume et al., 2011). To be used in tissue engineering, a scaffold should constitute a structure that promotes cell colonization and interactions, and the formation of an extracellular matrix (ECM), providing a structural support for the neoformed tissue (Raeisdasteh Hokmabad et al., 2017). Ideally, it should be porous, biocompatible, non-immunogenic and have appropriate mechanical properties suitable for the implant. Biomaterials commonly used for scaffold fabrication in tissue engineering include metals, ceramics and synthetic or natural polymers (Glowacki and Mizuno, 2008). Although synthetic polymers have several advantages (lack of immunogenicity, simple processing and controllable mechanical properties), natural materials, such as ECM, are naturally bioactive, biodegradable and are considered as surface ligands for cell adhesion (Guan et al., 2006; Lutolf et al., 2009). Recently, natural acellular ECM, derived from various tissues such as heart valves (Bloch et al., 2012; Moroni and Mirabella, 2014), blood vessels (Dall’Olmo et al., 2014; Mallis et al., 2020; Niklason and Lawson, 2020), skeletal muscle (Porzionato et al., 2015), placenta (Choi et al., 2013) and skin (Eweida and Marei, 2015), have been intensively studied as a “coating” for the surface of “scaffolds.” The ECM of these organs and tissues constitutes a rich reservoir of growth factors [as fibroblast growth factor 2 (FGF-2), vascular endothelial growth factor (VEGF), transforming growth factor-β (TGF-β)] (Taipale and Keski-Oja, 1997) and bioactive molecules like proteoglycans playing a role in cell adhesion, proliferation, differentiation and migration (Wight et al., 1992). However, since most of the natural ECM used in tissue engineering are derived from animals, plants or cadavers, problems of immunogenicity and transmission of pathogens are increasingly observed (Yang et al., 2004), which reduces their applications. In the present study, we focused on an ECM derived from neonatal human tissue (Wharton’s jelly from umbilical cord) that could be a very promising tool for regenerative medicine and tissue engineering fields (Dan et al., 2017b; Rosenbaum et al., 2008).

Wharton’s jelly (WJ) is a connective tissue found within the umbilical cord in which the two umbilical arteries and the umbilical vein are anchored. Until a few years ago, it was considered as a waste product and was discarded after birth. Very recently, interests in this tissue and its potential applications have been intensively investigated, and several teams have been working to better characterize it. This tissue, composed of different matrix proteins, is relatively gelatinous and has the function, among others, of protecting the umbilical vessels against compression and torsion (Ferguson and Dodson, 2009). WJ is essentially composed of an ECM containing mainly various types of collagen (types I, III, IV, and V), hyaluronic acid and several proteoglycans (mainly decorin), proteins that play a major role in cell adhesion (Nanaev et al., 1997; Gogiel et al., 2003). This tissue is also a source of growth factors such as FGF (fibroblast growth factor), IGF-1 (insulin growth factor-1), PDGF (platelet-derived growth factor), EGF (epidermal growth factor), and TGF-β (transforming growth factor beta 1) playing a major role in proliferation, cell differentiation, and the synthesis and remodeling of the ECM (Taipale and Keski-Oja, 1997; Sobolewski et al., 2005). In addition, WJ is easily isolated from the umbilical cord and is becoming increasingly central in several fields of application in tissue engineering and regenerative medicine (Dan et al., 2017b; Jadalannagari et al., 2017) such as cartilage regeneration (Xiao et al., 2017; Basiri et al., 2019) dermal regeneration (Beiki et al., 2017; Herrero-Mendez et al., 2017), vascular engineering (Dan et al., 2017a), etc.

Thus, because of all these aspects mentioned above, WJ has been of particular interest for several teams working in the field of tissue engineering. For example, the decellularized form of this tissue has been proposed as a 3D porous scaffold (Jadalannagari et al., 2017). The interest of this support has consisted in its bioactivity which can support and optimize cellularization, particularly in the context of cellularization by mesenchymal stromal cells (MSC), the cell type of choice in tissue engineering. This same principle has been studied in the promotion of hepatic differentiation of reprogrammed human cells (hiPSC) (Kehtari et al., 2019), or more recently in the context of regenerative therapy of intervertebral discs (Penolazzi et al., 2020).

The aim of the study is to propose an innovative biomaterial from human origin for tissue engineering applications. We therefore have developed a new and simple method to decellularize and extract matrix proteins from the human Wharton’s jelly tissue in order to obtain a solution of Wharton’s jelly extracellular matrix (WJ-ECMaa) that can be used as a surface coating improving cell adhesion, survival and proliferation. This study describes a decellularization and extraction protocol for a solution of WJ-ECMaa and validation of the decellularization efficiency. It aims to better characterize the biomaterial protein content by proteomic analysis and profile its 3D conformation by atomic force microscopy (AFM), a powerful multifunctional imaging platform (An et al., 2020). Its biocompatibility has been studied following several methods combining cell viability, proliferation and metabolic activity analysis. To validate its potential in vascular tissue engineering, its hemocompatibility has been performed by thrombin generation assay, a method offering a wide range of clinical and non-clinical applications (Binder et al., 2021).

## 2 Materials and Methods

### 2.1 Preparation of Wharton’s Jelly Extracellular Matrix

Human umbilical cords were obtained from full term, uncomplicated pregnancies, by successful vaginal route, and with the mother’s consent in accordance with the Cell Therapy Unit of the University Hospital Center (CHRU) of Nancy (Authorization number: TCG/11/R/011). Umbilical cords were stored in Hank’s Balanced Salt Solution (HBSS) at 4°C until reception and processing at the laboratory. After extensive washing of the umbilical cord with phosphate buffered saline (PBS), its amniotic membrane was cut longitudinally to expose and remove the umbilical vessels. Then, the WJ was carefully isolated without taking the outer membrane of the umbilical cord and cut into small pieces (3–5 mm^3^). The collected tissue was then washed three times with PBS and dry-frozen at −80°C. After slow defrosting (1°C/min), the WJ of three different umbilical cords was pooled to constitute one batch of WJ-ECMaa. Every pool of WJ was washed twice with 1% PBS followed by three washes under stirring for 5 min with sterile deionized ultrapure water. Two volumes of acetic acid were then added to one volume of WJ and homogenized for 10 min with an Ultra-Turrax homogenizer (Yellowline by IKA), on ice. The suspension was left under stirring overnight at 4°C and was finally centrifuged at 10,000 g for 30 min at 4°C. At the end of the centrifugation, the supernatant was collected and stored at −20°C. In this study, three different WJ-ECMaa pools (nine different umbilical cords) were analyzed, referred to as WJ-ECMaa A, WJ-ECMaa B, and WJ-ECMaa C.

### 2.2 Characterization of Wharton’s Jelly Extracellular Matrix Content

#### 2.2.1 DNA Quantification and Nucleic Acid Electrophoresis

In order to proceed to the determination of the nucleic acids contents in each sample, DNA was extracted from the different samples using a Quick-DNA™ Miniprep Plus Kit (D4068, ZymoResearch). A native WJ tissue from each extracted WJ-ECMaa was conserved as a control for decellularization. Thus, two sample processing protocols were applied according to the sample state: DNA extraction from tissue for the native WJ, and from biological fluid for the WJ-ECMaa. Briefly, DNA extraction from the native WJ was done as follows: 25 mg of WJ was placed in a microtube to which 95 µl of distilled water, 95 µl of Solid Tissue Buffer and 10 µl of Proteinase K were added. The tube was then vortexed for 15 s and incubated for 5 h at 55°C. The tube was then centrifuged for 1 min at 14,000 g and the supernatant volume was then transferred to a new tube. Two volumes of “Genomic Binding Buffer” were added and the tube content was then vigorously vortexed.

For DNA extraction from WJ-ECMaa, 200 µl of each WJ-ECMaa was placed in a microtube, to which 200 µl of Biofluid & Cell Buffer solution and 20 µl of proteinase K were added. The tube was then vortexed for 25 s and incubated for 10 min at 55°C; 420 µl of Genomic Binding Buffer was then added to the digested samples and the tube content was mixed vigorously.

After digestion, samples were transferred to Zymo-Spin™ IIC-XLR columns in a recovery tube and then centrifuged for 1 min at 12,000 g. The column was then placed in a new recovery tube and 400 µl of DNA Pre-Wash Buffer was added into the column. The tube was then centrifuged for 1 min at 12,000 g. Two washing steps were performed by adding, respectively, 700 and 200 µl of g-DNA Wash Buffer followed by a 1 min centrifugation at 12,000 g. DNA was eluted by adding 75 µl of DNA Elution Buffer and the mixture was incubated for 5 min and centrifuged for 1 min at 12,000 g. The total amount of isolated DNA was quantified using a Nanodrop One reading nanospectrophotometer (Thermo Scientific). The digested products were also analyzed by gel electrophoresis (0.8% [wt/vol] agarose in Tris–acetate–EDTA buffer) and visualized with GelRed (Biotium, Hayward, CA, United States).

#### 2.2.2 Relative Protein Quantification

WJ-ECMaa protein content was evaluated by a BiCinchoninic acid assay (BCA; UP40840A, Interchim). For preparation of the standard curve, bovine serum albumin (BSA; Sigma) was diluted to 2 mg/ml, then serial dilutions ranging from 0 to 2 mg/ml were prepared. Standards or samples (25 µl) were added in triplicate to a clear-bottomed 96-cell plate; 200 µl of the reagent solution (prepared by adding 1 volume of CuSO_4_ to 50 volumes of BCA solution) were added to each well. The plate was incubated for 30 min at 37°C in the dark. The optical density (OD) was measured at 570 nm using an automated plate reader (Varioskan Flash®, Thermo Fisher Scientific).

This test does not allow the determination of collagen, the main protein of the WJ-ECMaa. Indeed, this BCA assay is based on the optical detection of copper reduction mainly due to four amino acids which are cysteine or cystine, tyrosine and tryptophan (He, 2011). However, collagen is a triple-helix protein composed of repeated sequences of glycine followed frequently by proline or hydroxyproline (Prockop, 2004). Thus, the BCA assay shows a low affinity for the quantification of collagen present in a sample.

#### 2.2.3 Collagen Quantification

The collagen content of the ECM was quantified with a Total Collagen Assay Kit (perchlorate-free; ab222942, Abcam) and determined according to a standard curve. ECM samples were hydrolyzed by adding 10 N NaOH solution (1 v/1 v) for 1 h at 120°C. After cooling on ice, 10 N HCl solution was added (1 v/1 v/1 v) to neutralize the pH of the solution. Samples were then centrifuged for 5 min at 10,000 g and supernatants were collected; 10 µl of supernatants was placed in a clear-bottomed 96-well plate and incubated at 65°C in order to evaporate the extraction solution. The remaining crystals of each well were dissolved by adding 100 µl of Oxidation Reagent Mix (6 µl of chloramine T + 94 µl of oxidation buffer) for 20 min at room temperature. Developer (50 μl) was added to each reaction well and the plate was incubated at 37°C for 5 min. DMAB Concentrate Solution (50 µl) was added to each well and the plate was incubated for 45 min at 65°C. The OD of each well was measured at 560 nm using an automated plate reader (Varioskan Flash, Thermo Scientific, France).

#### 2.2.4 Glycosaminoglycans Quantification

The GAG content of the extracted ECM was quantified using a spectrophotometric method and determined according to a standard curve. Standard dilutions were prepared by serially diluting a standard solution of chondroitin-6-sulfate (C4384-1G, Sigma) to 1 mg/ml with Tris buffer (50 mM, pH 8) in order to obtain these different concentrations: 5, 10, 20, 40, 60, 80, 100, and 200 μg/ml. To prepare samples, 1 ml of each ECM was dried for 48 h in an incubator at 56°C. They were then digested overnight at 60°C in a 200 µl buffer solution [20 mM NA_2_HPO_4_ (28028.298, VWR), 1.4 mM EDTA (ED-5K, Sigma), 0.2 mM dithiothreitol (DTT; 43819-1G, Sigma)] to which was added 10 µl of a papain solution (6 mg/ml; P4762-100 MG, Sigma). The reaction was stopped with 10 µl of 0.22 M iodoacetic acid solution (L328074 009, Merck) and 380 µl of Tris buffer (50 mM, pH 8). A 20 µl aliquot of each sample preparation and standard were placed in triplicate in a clear-bottomed 96-well plate. After adding 250 µl of Goldberg solution [made by dissolving 32 mg of 1,9-dimethylmethylene blue (DMB; 23481-50-7, Polysciences Inc.) in 5 ml of ethanol] to each well, the OD of each well was measured at 525 nm using an automated plate reader (Varioskan Flash, Thermo Scientific, France).

#### 2.2.5 Mass Spectrometry Analysis

Aliquots (10 µg) of each WJ-ECMaa acidic batch were used. Triplicate aliquots were analyzed for each pool. Volumes were equilibrated to 10 µl in 0.5 M acetic acid. Pepsin (100 ng; Sigma) was added to allow overnight digestion at 4°C. Thereafter, samples were dried for 2 h in a speed-vac evaporator (Eppendorf® Concentrator Plus), resuspended in 10 µl of 6 M urea, 50 mM Tris, pH 8.0 and processed as follows: cysteine residues were reduced by addition of 4 mM DTT for 45 min, then alkylated by addition of 40 mM iodoacetamide (IAA) for another 45 min. The IAA reaction was blocked by adding another portion of 40 mM DTT for 10 min. Then 90 µl of 100 mM Tris and 10 mM CaCl_2_ were added together with 1/40th (weight trypsin/weight protein extract) sequencing-grade trypsin (Promega) and digestion was allowed to proceed overnight at 37°C. Samples were acidified by addition of 5 µl of 10% trifluoroacetic acid. Samples were fractionated by nanoHPLC on an Ultimate3000 system equipped with a 20 µl sample loop, a pepMap 100 C18 desalting precolumn and a 15 cm pepMap RSLC C18 fractionation column (all from Dionex). Samples (5 µl) were injected using the µl pickup mode and eluted by a 22%–45% ACN gradient over 30 min at 300 nl/min. Fractions (226, 6 s each) were collected on a ProteineerFcII (Bruker) over 22.6 min and eluted fractions were directly mixed on MTP-1536 BC target (Bruker) spots with α-cyano-4-hydroxycinnamic acid (Bruker). LC-MALDI runs dedicated to peptide relative quantification were processed using dedicated automatic methods piloted by WARP-LC software on an Autoflex speed MALDI-TOF/TOF mass spectrometer (Bruker), first in MS mode in the 700–4,500 mass range, using next-neighbor external calibration for all MALDI spots. Relative quantification was performed from the MS analysis using ProfileAnalysis software (Bruker) with time alignment and quantile normalization, requiring a minimal bucket value of 3, to highlight significant changes between independent extracts (fold changes above 2, t-test *p*-value < 0.05) and generate the corresponding Scheduled Precursor Lists. Thereafter, masses exhibiting a signal/noise ratio above 20 were processed for MS/MS analysis in LIFT mode. Peptide assignments were performed from TOF/TOF spectra by Mascot interrogation (Matrix Science) of the whole human database piloted in Mascot and compiled by Proteinscape with a mass tolerance of 50 ppm in TOF mode and 0.8 Da in TOF/TOF mode, with optional cysteine carbamidomethylation, methionine and proline oxidation, and trypsin cut. The minimal Mascot score for peptides was set to 20 and that for proteins was set to 80. Results were cross-validated by interrogating an irrelevant database using the same criteria. Relative quantification data were exported to Proteinscape with the following alignment criteria: maximal time deviation, 2 min; maximal mass deviation, 0.03 Da. Protein change calculations were allowed from a minimum of two peptides passing relative quantification quality control as defined in ProfileAnalysis. A chart pie regarding biological process is provided as Supplementary Figure S1.

### 2.3 Evaluation of the Wharton’s Jelly Extracellular Matrix Coating

#### 2.3.1 Fluorescent Labelling of Primary Amine Residues of the Wharton’s Jelly Extracellular Matrix Deposit

The WJ-ECMaa coating was evaluated by binding and staining the primary amine groups of ECM proteins using NH_2_-sulfo-NSS-biotin (EZ-Link™ Sulfo-NHS-SS-biotin, 21331, Thermo Scientific). First, the WJ-ECMaa was incubated at 4°C overnight under stirring with NH_2_-NSS-biotin (at a concentration of 0.48 mg/ml). The link reaction was stopped by adding 50 mM Tris-Base solution. Labeled WJ-ECMaa (400 µl) was then deposited into triplicate wells in a clear-bottomed 24-well plate for 24 h at 37°C. Each well was washed with deionized ultrapure water and incubated for 30 min at room temperature with Invitrogen streptavidin–AlexaFluor™ 488 conjugate (S11223, Thermo Fisher Scientific) at 1/200 dilution. After incubation, the coatings were washed with deionized water before imaging with epifluorescence microscopy (Leica DMI 3000B microscope). For confocal imaging, WJ-ECMaa was deposited on glass slides. Samples were observed with a Leica TCS SP5 X microscope.

#### 2.3.2 Characterization of the Surface Coating by Atomic Force Microscopy

Before each measurement, fresh samples deposited on glass slides were intensively rinsed with Milli-Q water and then slowly and completely dried with nitrogen. AFM experiments were carried out using an MFP-3D-BIO instrument (Asylum Research Technology, Atomic Force F&E GmbH, Mannheim, Germany). MLCT silicon nitride cantilevers used for these experiments were purchased from Bruker (Bruker France SAS, Palaiseau, France). Experiments were performed in air at room temperature and in contact mode. All images were collected with a resolution of 512 × 512 pixels at a scan rate of 1 Hz.

### 2.4 Cytocompatibility Assays

The cytocompatibility of the WJ-ECMaa was evaluated with human mesenchymal stromal cells derived from the Wharton’s jelly (WJ-MSC) cultured on this coating for 12 days with medium renewal every 2 days. In order to compare the results obtained, a control condition was carried out consisting of a coating of collagen which is the most used ECM protein for cell culture (Collagen Type I Rat Tail, 354236, Corning). Briefly, to obtain this control coating, 400 µl of collagen at a concentration of 0.1 mg/ml was placed in a 24-well culture plate in triplicate. The plate was then incubated at 37°C for 1 h before complete removal of the solution. Wells were intensively washed with 1 × PBS before cell seeding.

#### 2.4.1 Wharton’s Jelly Extracellular Matrix Isolation and Culture

WJ was also used in this study for its important content of MSC, immune evasive cells showing an important potential for tissue engineering applications with interesting immunomodulation properties (Weiss et al., 2008; El Omar et al., 2014; Li and Hua, 2017; Mallis et al., 2021). WJ-MSC were mechanically collected by applying the explant method based on the ability of cells to migrate out of the tissue and adhere to the culture surface. Briefly, the WJ was isolated as mentioned above, then it was cut into approximately 5 mm pieces that were placed in a six-well culture plate and cultured at 37°C in a humidified atmosphere containing 5% (v/v) CO_2_ in a complete medium: α-MEM (BE12-169F, Lonza) supplemented with 10% fetal bovine serum (FBS; lot S132507, Dutscher), Fungizone (100 mg/ml; 11520496, Fischer), penicillin (100 IU/ml; P0781, Sigma) and L-glutamine (200 mM; G7513, Sigma). The culture medium was changed every 3 days until day 15 when WJ explants were removed from wells. hWJ-MSC were then trypsinized for 5 min at 37°C (trypsin–EDTA 0.05%, Gibco), counted and seeded at a concentration of 1 × 10^5^ cells/cm^2^ in a T75 cell culture flask. Cells at that stage are at the first passage (P1). When reaching a confluence of approximately 90%, the trypsinization step is repeated until cells reach the third passage. The phenotype of the P3 WJ-MSC used in this study was confirmed by flow cytometry according to a panel of several positive and negative surface antigens (cluster of differentiation, CD) described for MSC, including: positive expression of CD90, CD73, CD44, and CD105 and a lack expression of CD45, CD34, HLA-DR, and CD86 (Dominici et al., 2006) (These results are available in Supplementary Figure S2).

#### 2.4.2 Cell Metabolic Activity

The metabolic activity of WJ-MSC was assessed by MTT [3-(4,5-dimethylthiazol-2-yl)-2,5-diphenyltetrazolium bromide] assay. Briefly, cells were seeded at a density of 1.5 × 10^3^ cells/cm^2^ onto a 24-well plate coated with WJ-ECMaa. After 1, 7, and 12 days of culture, the 2 mg/ml MTT solution (thiazolyl blue tetrazolium bromide; M2128-5G, Sigma) was added to the wells containing 100 μl of α-MEM medium and cells were incubated for 4 h at 37°C. The medium was then carefully removed, and formazan crystals were dissolved with 200 μl of dimethylsulfoxide (DMSO) for 5 min. A 100 μl aliquot from each well was transferred to a 96-well plate in duplicate and the OD was measured at 550 nm using an automated plate reader (Varioskan Flash, Thermo Scientific, France).

#### 2.4.3 Coating Cytotoxicity

The potential cytotoxicity of the coating was evaluated by measuring the lactate dehydrogenase (LDH) released by WJ-MSC cultured on WJ-ECMaa using an LDH assay Cytotoxicity Detection Kit^plus^ (04744934001, Roche) on hWJ-MSC at 1, 7, and 12 days. Cells were seeded at a density of 1.5 × 10^3^ cells/cm^2^ onto a 24-well plate previously coated with WJ-ECMaa. At each time point, triplicates of 100 µl of each well were transferred to a 96-well plate containing 100 µl/well of the reaction solution (prepared by mixing 1 volume of catalyst with 45 volumes of dye solution). After 20 min of incubation under stirring in the dark at room temperature, the absorption was measured at 490 nm using an automated plate reader (Varioskan Flash, Thermo Scientific, France). For each condition, an additional well was used as a positive control for cytotoxicity. Cells in the control well were lysed by incubation at 37°C for 10 min with 20 µl of Triton™ X-100 (T8787-50 ml, Sigma) before the cytotoxicity analysis. The percentage toxicity was then calculated for each condition and normalized to the percentage obtained in the collagen coating condition.

$$\left(1-\left(\frac{X_{ECM}}{X_{ECM\;Death}}-\frac{X_{Collagen}}{X_{Collagen\;Death}}\right)\right)\;\text{∗}\;100$$ (1)

#### 2.4.4 Cell Proliferation

In parallel, at each time point, the total DNA of each sample was collected and quantified using a Hoechst 33258 fluorescent dye assay (H3569, Invitrogen, France). Briefly, WJ-MSC were cultivated on WJ-ECMaa coating in a 24-well plate. The culture medium was removed and cells were lysed by three freeze–thaw cycles (liquid nitrogen to 37°C water bath) in 100 µl of Hoechst buffer (Tris-base (10 mM); EDTA (1 mM); NaCl (0.1 M, pH 7.4). Hoechst solution (900 μl, 0.1 μg/ml) was then added to each well. Samples were then placed in triplicate in a 96-well microplate for a fluorescence-based assay with a volume of 300 µl for each well. The fluorescence was measured with a microplate fluorescence reader (Varioskan Flash, Thermo Scientific, France) at excitation/emission wavelengths, respectively, of 356 and 458 nm. The mass of DNA was calculated in µg by extrapolation from an 8-point standard curve generated with calf thymus DNA (D3664, Sigma).

### 2.5 Evaluation of Wharton’s Jelly Extracellular Matrix Hemocompatibility by Thrombin Generation Assay

Thrombin is a key enzyme of the coagulation cascade. Its measurement will give direct information about the thrombogenicity of the biomaterial. This evaluation is required according to ISO 10993-4/2017. The study protocol was approved by the local ethics committee (Comité de Protection des Personnes, agreement 15/10/2014). All study patients provided written informed consent. A 96-well plate was coated with WJ-ECMaa as described above. After rinsing the wells thoroughly with 1 × PBS, 10 µl of HEPES saline buffer (HBS) was added to each well, as well as 40 µl of human plasma (pool from three different donors) and a home-made mixture of phosphatidyl-choline/serine/ethanolamine (60/20/20, mol%) (Avanti Polar Lipids, United States) at a final concentration of 4 μmol/L. Coagulation was triggered by adding recombinant human tissue factor (Innovin®, Dade Behring) at 1/30,000 dilution, and thrombin generation was monitored by quantification of a fluorescent substrate specific to thrombin Z-Gly-Gly-Arg-AMC (Bachem, Bubendorf, Switzerland) at a final concentration of 417 μmol/L, in a Fluoroskan Ascent fluorometer (Thermo LabSystem). Thrombin generation was measured every 15 s for 50 min at excitation and emission wavelengths of 390 and 460 nm, respectively. Results were then analyzed in comparison with the same pool of plasma used as a control, with the dedicated software (Thrombinoscope BV, Maastricht), and expressed as the endogenous thrombin potential (ETP, corresponding to the area under the generation curve), peak (the maximal value of generated thrombin concentration) and velocity index (the effective rate of thrombin generation between lag time and time to peak).

### 2.6 Evaluation of the Wharton’s Jelly Extracellular Matrix Coating on Biological Tissue

Three human umbilical arteries of 20 cm of length were cut into two equal pieces to form two groups for each artery: artery coated with WJ-ECMaa Vs. Uncoated artery. Each segment was prepared separately as follows: “uncoated segments” were incubated in supplemented α-MEM at 37°C until the cellularization step. In parallel, each “coated segment” was placed under stirring into 10 ml of WJ-ECMaa for 24 h at 37°C. Segments were then thoroughly rinsed (externally and internally by perfusion) by three wash steps with complete α-MEM medium for 10 min at 37°C under stirring. Each segment (uncoated and coated) was then placed into a 50 ml Falcon tube filled with complete α-MEM medium in which 700.000 WJ-MSC (at P3) previously labelled with a lipophilic membrane stain DiI (Invitrogen, D282) were added. This staining was performed by incubating cell suspension at a concentration of 1.000.000 cells/ml for 5 min at 37°C with 25 µl of DiI per ml. The adhesion step was performed overnight at 37°C under rotation and adhesion efficacy was evaluated by epifluorescence microscopy (at day 1). Cellularized segments were then placed into T75 flasks and the medium was renewed three times per week. A cellularization follow-up was performed by epifluorescence microscopy at days 2, 5, and 7 and by epifluorescence macroscopy at day 9. Before observation, segment was cut longitudinally to expose the luminal surface.

### 2.7 Statistical Analyses

Statistical analysis of data was performed using GraphPad Prism Version 8. Normality was verified by the Shapiro–Wilk test. Normally distributed data were then analyzed using one-way analysis of variance (ANOVA) with Tukey’s post hoc test, two-way ANOVA with Bonferroni post hoc test, or unpaired t-test.

Differences between the groups with a probability value higher than 95% (*p* < 0.05) were considered statistically significant.

## 3 Results

This study aimed to characterize a new ECM for potential use as a natural surface biological coating in the field of tissue engineering. The originality of this biomaterial is its human origin combined with easy, short and inexpensive extraction from umbilical cords.

The protocol applied on WJ to obtain the WJ-ECMaa is easily reproducible and is composed of two major steps. First the WJ was decellularized by SDS baths and washed several times. Proteins contained in the connective tissue were then extracted with acetic acid. This extraction protocol allows the solubilization and decellularization of the ECM from WJ and its use as a biocompatible surface coating promoting cell adhesion, viability and proliferation.

### 3.1 Wharton’s Jelly Extracellular Matrix DNA Content

As the WJ-ECMaa is intended to be used as a biomaterial for tissue engineering applications, its extraction’s protocol must allow an efficient decellularization in order to prevent an immune rejection.

**FIGURE 1:**
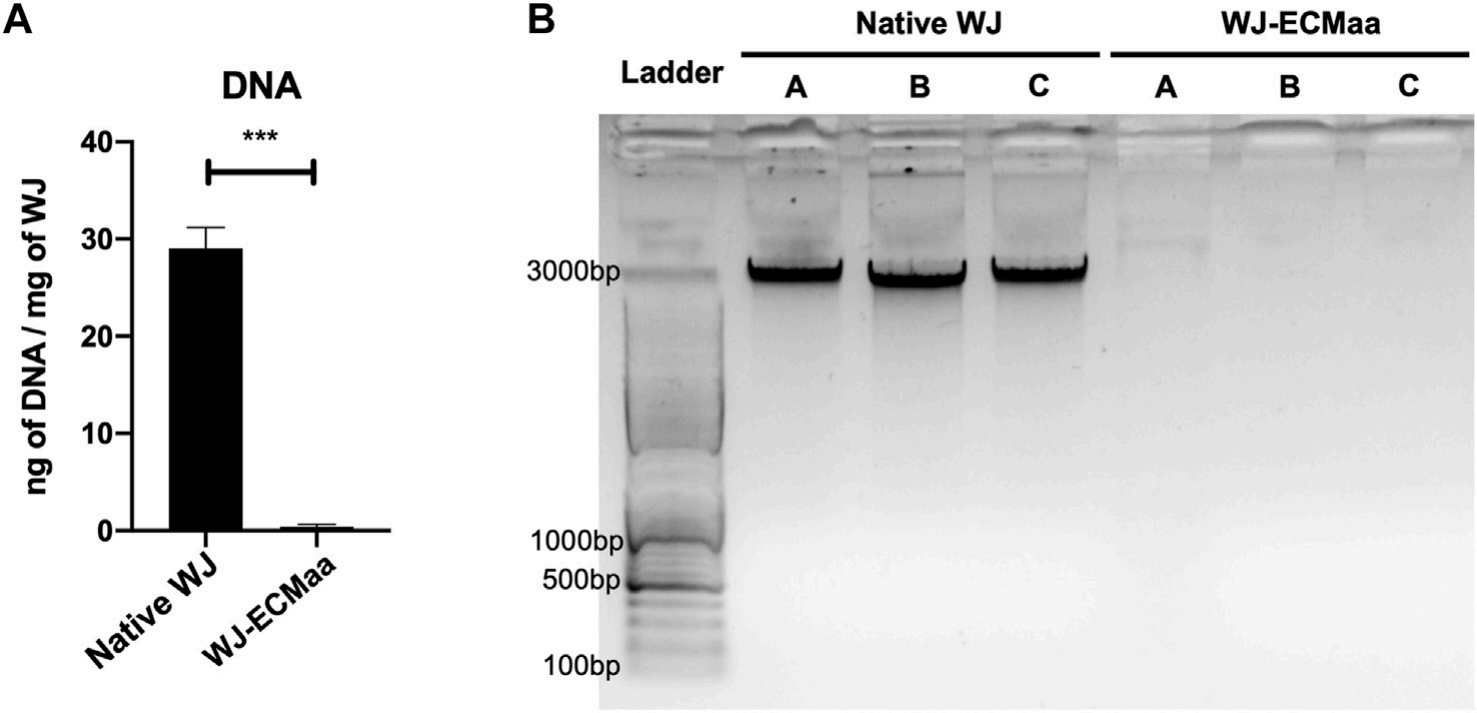
Evaluation of the decellularization efficiency of WJ-ECMaa. **(A)** DNA quantification in native WJ and decellularized WJ-ECMaa. Results represent the mean of three different assays. **(B)** Nucleic acid migration profile for the three native WJ and their respective decellularized WJ-ECMaa. Results are presented as the mean ± SEM for each condition, and statistically analyzed following an unpaired t-test (*p*-value: ***< 0.001); *n* = 3.

The DNA content of the different WJ-ECMaa was quantified, and the results were compared to the amount of DNA found in the native tissue before extraction (Figure 1). An average of 29 ng of DNA was identified for 1 mg of native WJ. This value decreased drastically in the decellularized extracted ECM, in which an average of 0.424 ng of DNA was found for 1 mg of WJ (Figure 1A). This result was confirmed by nucleic acid electrophoresis showing the absence of nucleic material in the three extracted WJ-ECMaa compared to their original native tissue (Figure 1B).

### 3.2 Wharton’s Jelly Extracellular Matrix Protein Content

WJ-ECMaa extracted in a liquid form has not yet been described in the literature, thus it is essential to characterize its content to identify its potential properties and possible use as a biomaterial.

**FIGURE 2:**
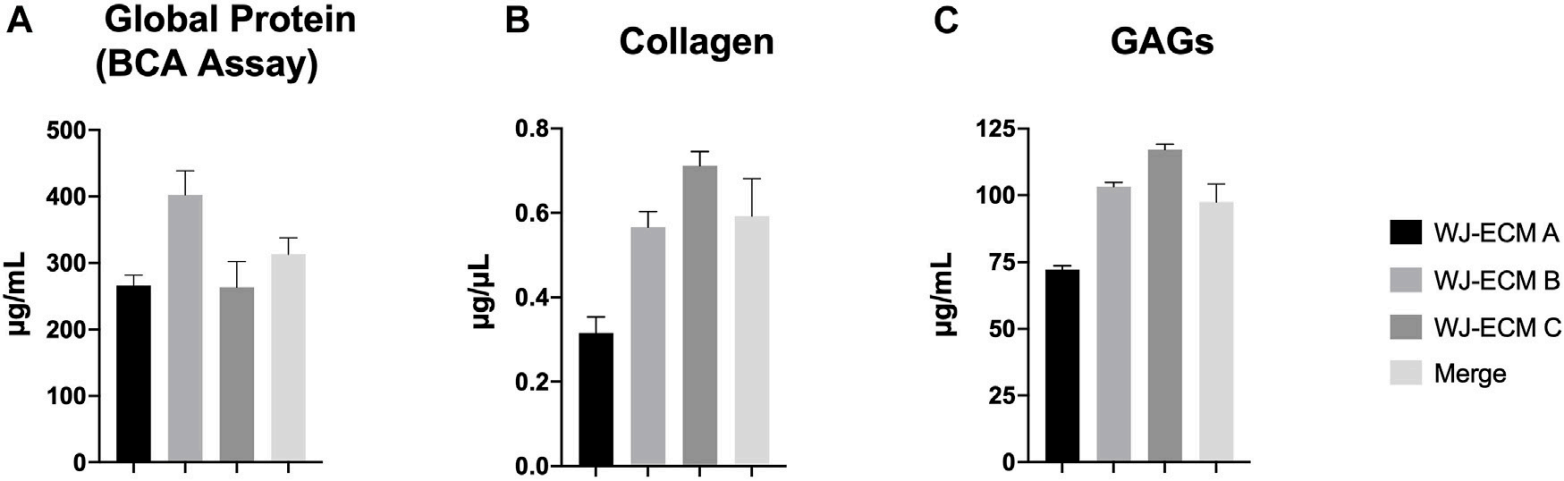
Global protein, collagen and glycosaminoglycan (GAG) content of WJ-ECMaa. **(A)** Protein content for each pool of WJ-ECMaa and the corresponding mean values. **(B)** Collagen content for each pool of WJ-ECMaa and the corresponding mean value. **(C)** GAG content for each pool of WJ-ECMaa and the corresponding mean value. Results are presented as the mean of the three pools of WJ-ECM ± SEM.

To best characterize the protein content of the three pools of WJ-ECMaa, a first general protein quantification was performed using a BCA assay, allowing determination of the global protein content by quantification of cysteine or cystine, tyrosine and tryptophan residues. Thus, this assay strongly underestimates the collagen content which is principally composed of glycine, proline and hydroxyproline (Prockop, 2004; He, 2011).Thus, the three pools of ECM in our study contained an average of 309.1 μg/ml of protein (A: 260.2 μg/ml; B: 402.4 μg/ml; C: 264.6 μg/ml) (Figure 2A). The dosage of soluble collagen allowed us to obtain an average collagen concentration of 0.592 μg/μl (A: 0.316 μg/μl; B: 0.749 μg/μl; C: 0.712 μg/μl) (Figure 2B). Moreover, analysis of the content of GAGs in each ECM indicated their presence at an average concentration of 97.53 μg/ml (72.18, 103.3, and 117.1 μg/ml for each of the different matrices, respectively) (Figure 2C).

To better characterize the protein profile of the WJ-ECMaa, three aliquots from each pool of WJ-ECMaa were analyzed by mass spectrometry. The results show that for the three WJ-ECMaa pools, the main protein extracted from WJ was collagen, with a predominance of type I followed by type III. Other proteins such as lumican, decorin, dermatopontin, and transgelin were also identified, but to a far lesser extent (Table 1).

**TABLE 1:** Protein content identified by mass spectrometry analysis of the WJ-ECMaa pool. Results represents the mean of the values obtained for the three pools of WJ-ECMaa.

Row	Accession	Scores	Protein	Molecular weight (kDa)	#Peptides
1	CO1A1_HUMAN	4,696.0	Collagen alpha-1(I) chain	138.9	87
2	CO1A2_HUMAN	3,123.9	Collagen alpha-2(I) chain	129.2	54
3	CO3A1_HUMAN	2,571.3	Collagen alpha-1(III) chain	138.5	46
4	ACTH_HUMAN	277.9	Actin. gamma-enteric smooth muscle	41.8	4
5	LUM_HUMAN	212.6	Lumican	38.4	5
6	ACTB_HUMAN	158.6	Actin. cytoplasmic 1	41.7	3
7	ALBU_HUMAN	133.5	Serum albumin	69.3	4
8	IGHG1_HUMAN	87.9	Ig gamma-1 chain C region	36.1	3
9	CO6A3_HUMAN	85.4	Collagen alpha-3(VI) chain	343.5	2
10	DERM_HUMAN	41.5	Dermatopontin	24.0	1
11	TAGL_HUMAN	40.2	Transgelin	22.6	1
12	PGS2_HUMAN	37.8	Decorin	39.7	1

Principal component analysis of the mass did not suggest significant differences between the three ECM (nor between replicates if all variables are considered). The quantitative analysis highlights some proteins that vary between the three pools, notably serum albumin (data not shown).

### 3.3 Wharton’s Jelly Extracellular Matrix Coating Observations

The WJ-ECMaa has been designed to be used as surface coating to optimize cellularization step in a context of tissue engineering, but it also could be used for cell culture applications. Thus, it was important to characterize the surface of the coating namely the disposition of the fibers, the appearance of collagen fibers and the thickness of the coating. This characterization may help predict the WJ-ECMaa cell interactions. WJ-ECMaa coating was analyzed by fluorescent observations and by atomic force microscopy.

Labeling of the primary amine residues of WJ-ECMaa proteins allowed the visualization and characterization of the deposit of the matrix used as a 2D surface coating.

**FIGURE 3:**
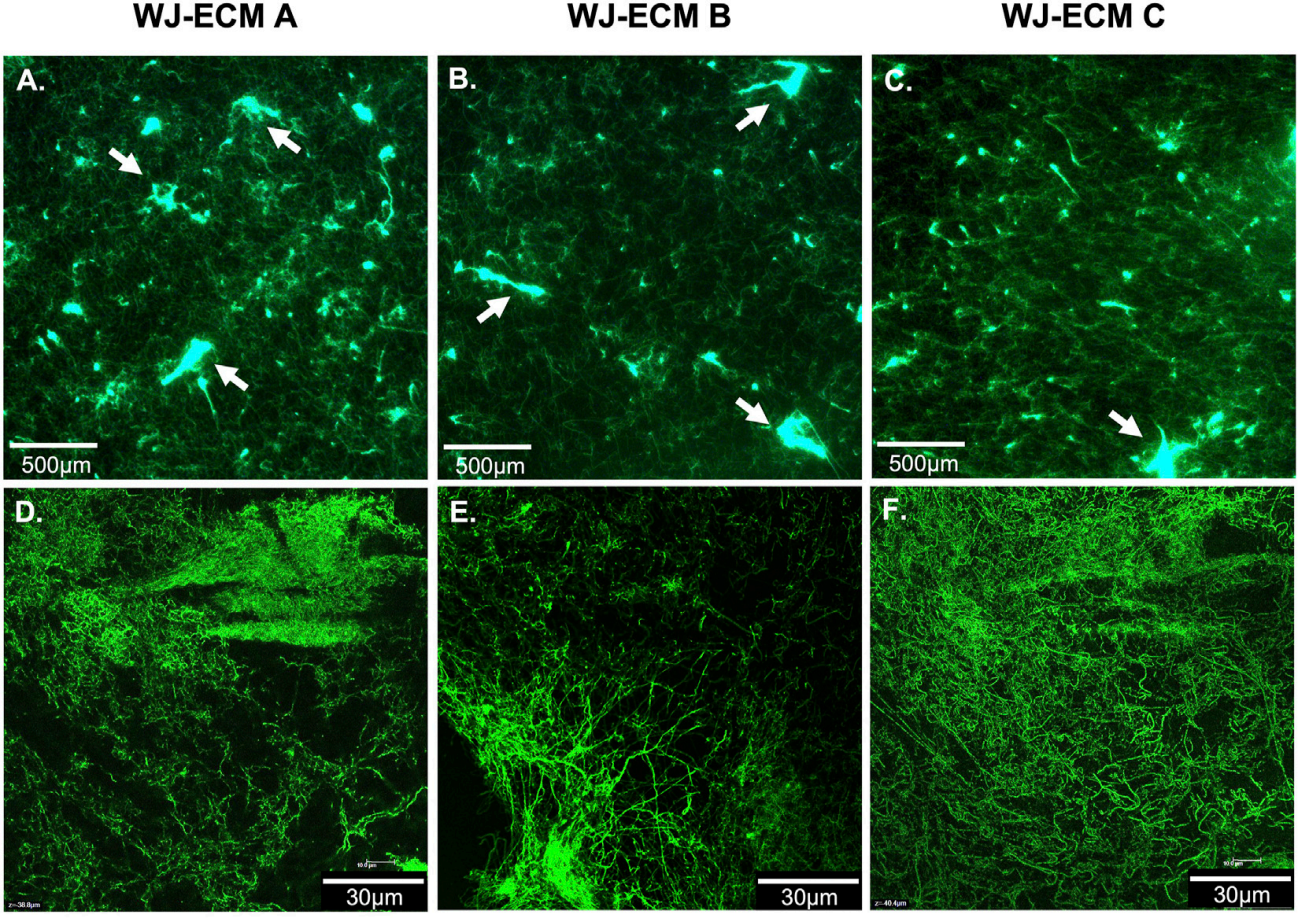
Microscopic observations of WJ-ECMaa coating. **(A–C)** Epifluorescence images of WJ-ECM A, WJ-ECM B, and WJ-ECM C, respectively (aggregates are indicated with white arrows). **(D–F)** Confocal microscopy observations of WJ-ECM A, WJ-ECM B, and WJ-ECM C coating, respectively.

The WJ-ECMaa coating formed a grid over all the observed surfaces as shown by epifluorescence microscopy observations. The resulting deposit was observed as a dense network of heterogeneous fibers with some aggregates (Figures 3A–C). These observations were verified by observation at higher resolution by confocal microscopy (Figures 3D–F). Although the overall shape of the deposit was identical for all three pools of WJ-ECMaa, differences were noted, especially for the deposit of WJ-ECM C which showed a denser distribution of fibers.

**FIGURE 4:**
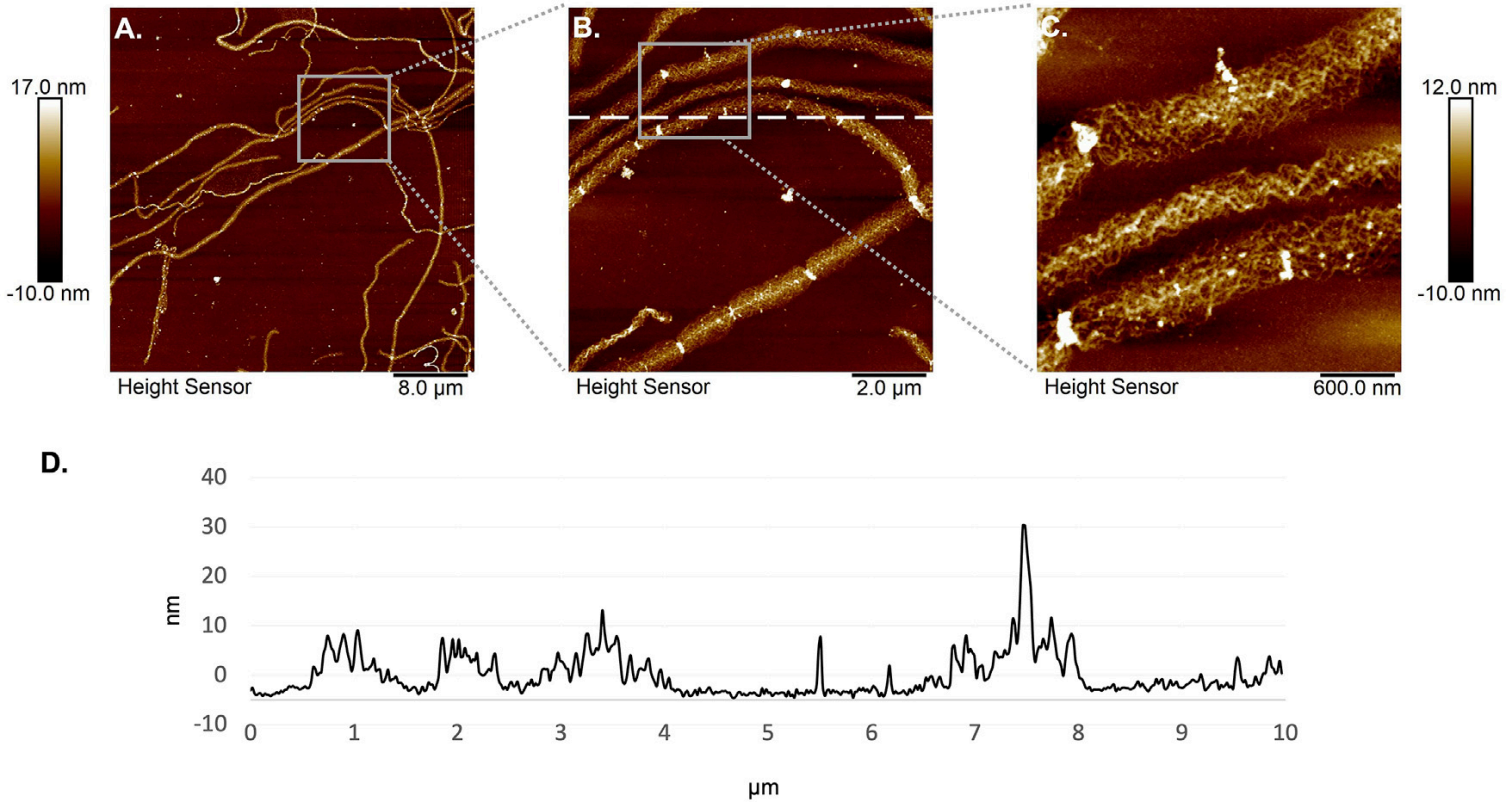
Atomic force microscopy observations of fibers composing the WJ-ECMaa. **(A–C)** Height sensor acquisitions of fibers present in the WJ-ECMaa coating. **(D)** Cross-section analysis of height sensor acquisition.

The optically observable fibers found in the WJ-ECMaa deposit were heterogeneously interconnected varying in the diameter and length of the fibers, as well as in the density of the resulting network. AFM analysis, allowing a better resolution observation, showed that they were structured in the same way as depicted in Figure 4. Fibers were around 10–20 nm in height, 0.2–1.0 µm wide for length greater than 50 µm. Besides, these fibers showed asperities or aggregate spot along them with size of up to 40 nm as illustrated by transversal cross-section (Figure 4D). At higher resolution, the specific internal structure of these fibers can be observed. They seem to be the assembly of several smaller fibers arranged in a solenoidal-like or triple-helical manner along their length as reported herein (Figure 4C).

### 3.4 Cytocompatibility of Wharton’s Jelly Extracellular Matrix Coating

The WJ-ECMaa coating biocompatibility was then evaluated using MSC derived from the WJ. Several experiments were carried out over a period of 12 days, to determine the coating potential cytotoxicity, its impact on the cell metabolic activity and proliferation.

**FIGURE 5:**
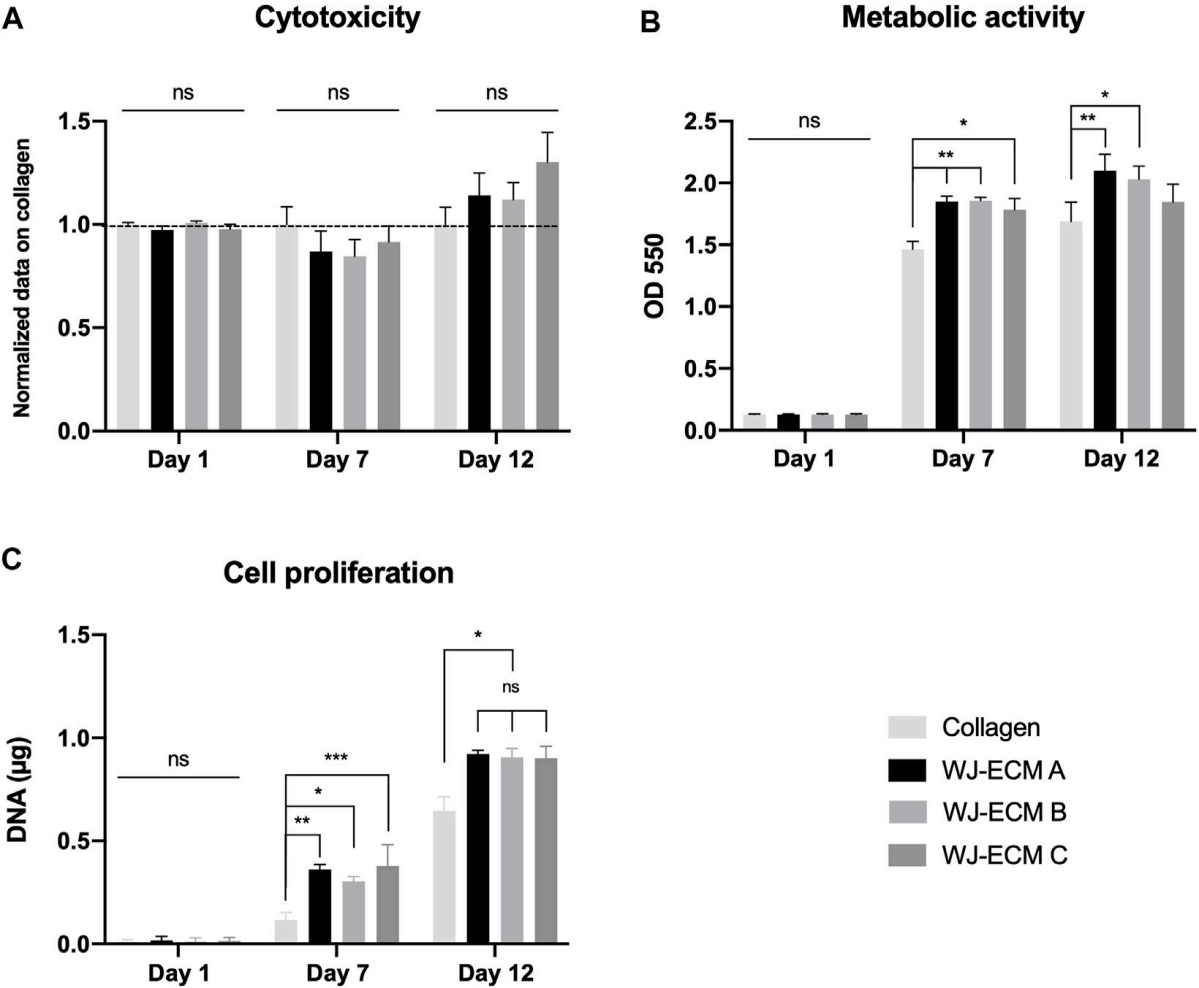
Study of the cytocompatibility of WJ-ECMaa coating with h-WJ-MSC. WJ-MSC were seeded and expanded on WJ-ECMaa and collagen coatings, and cell behavior was evaluated at 1, 7, and 12 days of culture. **(A)** Cytotoxicity of the coating was evaluated by release of LDH by cells. For each day, results were normalized to collagen-induced cytotoxicity. **(B)** Metabolic activity of WJ-MSC observed at days 1, 7, and 12 according to the coating. **(C)** Monitoring of the cell proliferation rate by DNA quantification at days 1, 7, and 12 according to the seeded coating. Results are presented as the mean ± SEM for each condition, and were statistically analyzed following two-way ANOVA (*p*-value: *< 0.05, **< 0.01, ***< 0,001); *n* = 3.

To examine the cytocompatibility of the WJ-ECMaa coating, human WJ-MSC viability, mitochondrial activity and proliferation on this surface were analyzed for a culture period of 12 days. The results obtained were compared to a coating control condition consisting of a commercially available collagen I (0.1 mg/ml), frequently used as a support for cell adhesion and proliferation (Figure 5).

To evaluate the potential cytotoxicity of the WJ-ECMaa coating, LDH assays were performed on WJ-MSC seeded on this surface at 1, 7, and 12 days of culture (Figure 5A). For each day, cytotoxic results were analyzed and normalized to those obtained with cells seeded on the collagen I coating. For days 1 and 7, normalized results obtained for the LDH released by cells seeded on the three different pools of WJ-ECMaa showed no significant differences from the control condition. Moreover, no differences in the cytotoxicity results were detected between each of the three WJ-ECMaa. Even if not significant, at day 12, cytotoxicity results for cells seeded on WJ-ECMaa coatings showed an increasing trend compared to those obtained with the control condition (1.14 ± 0.10, 91.12 ± 0.08, and 1.30 ± 0.15 for WJ-ECM A,B,C, respectively).

An MTT assay was also performed to check if the WJ-ECMaa had an impact on the metabolic activity of the cells seeded on this coating compared to cells seeded on collagen I coating (Figure 5B). At day 1, no significant differences in mitochondrial activity were found between each of the different conditions. At day 7, the OD recorded for each of the WJ-ECM A, B and C conditions as well as for collagen was 1.85 ± 0.04, 1.86 ± 0.03, 1.78 ± 0.09, and 1.46 ± 0.06, respectively. These values reflect a significantly higher metabolic activity for cells deposited on the different WJ-ECMaa compared to those seeded on the collagen control. Cell metabolic activity increased mildly at day 12 for all conditions, with significantly higher values for cells seeded on WJ-ECM A and B coatings compared to the control condition. The metabolic activity of the MSC deposited on WJ-ECM C was not significantly different from that for the control condition (*p*-value of 0.58) although a tendency to increase was notable.

On the other hand, in terms of proliferation, at day 7, cells seeded on WJ-ECMaa showed significantly higher proliferation rates compared to the control condition (Figure 5C). At day 12, results were homogeneous for the three pools of ECM, indicating that WJ-MSC seeded on the WJ-ECMaa have improved proliferation compared to those cultured on collagen, with a mean value of 0.910 ± 0.066 µg for WJ-ECM conditions (A: 0.92 ± 0.02 µg; B: 0.91 ± 0.04 µg; C: 0.90 ± 0.06 µg) while 0.645 ± 0.069 µg of DNA was quantified for cells seeded on the collagen coating. These results explain the high values of LDH released by the cells seeded on the WJ-ECMaa at day 12 since the cells had a higher proliferation rate under these conditions. Thus, they reached confluence more quickly and apoptosis could be induced.

### 3.5 Hemocompatibility of Wharton’s Jelly Extracellular Matrix

The mechanically extracted WJ-ECMaa is intended to be used as a biomaterial surface coating so it can be in direct contact with the blood. Thus, it is crucial to evaluate its hemocompatibility.

**FIGURE 6:**
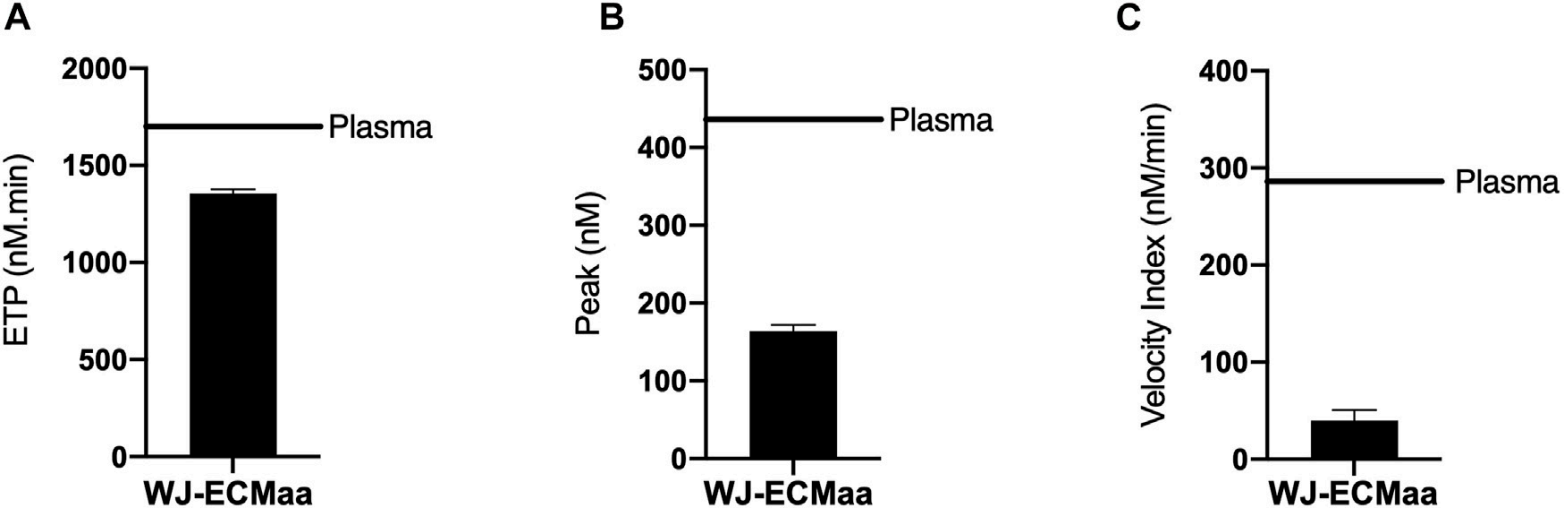
Thrombogenicity evaluation of the WJ-ECMaa coating monitored by thrombin generation assay. **(A)** ETP: endogenous thrombin potential, a reflection of the thrombogenic potential of the sample ; **(B)** Peak: maximum value of active thrombin generated with the sample; **(C)** Velocity index: reflecting the slope of the explosive thrombin generation. Results presented as mean ± SD.

WJ-ECMaa coatings were subjected to thrombin generation tests. Results were compared to those obtained in conditions where only the plasma used for the experiment was tested (Figure 6). Thus, the values of ETP, maximum peak and velocity index observed for the reference plasma were 1,707 nM min, 436 and 287 nM/min, respectively. The results show that the WJ-ECMaa coating has a good hemocompatibility profile with an anticoagulant behavior compared to the control condition, with an ETP of 1,356 ± 74 nM min, a maximum peak value of 164 ± 27 nM and a velocity index of 40 ± 11 nM/min (Figure 6B).

### 3.6 A Vascular Tissue Engineering Application: WJ-MSC Coating on a 3D Biological Tissue

To complete this study, a proof of concept has been carried out to observe the WJ-ECMaa coating impact on cellularization of a 3D tissue. As the research of our team is in the field of vascular engineering, we decided to apply this coating on the luminal surface of decellularized umbilical arteries and to study its effect on the cellularization with WJ-MSC of this same surface. MSC have been used for the growing evidence of their important potential in tissue engineering and were preferred to mature vascular cells requiring invasive extraction, are inherently heterogeneous, dedifferentiate following prolonged culturing *in vitro* and are not compatible for an allogeneic context (El Omar et al., 2016).

**FIGURE 7:**
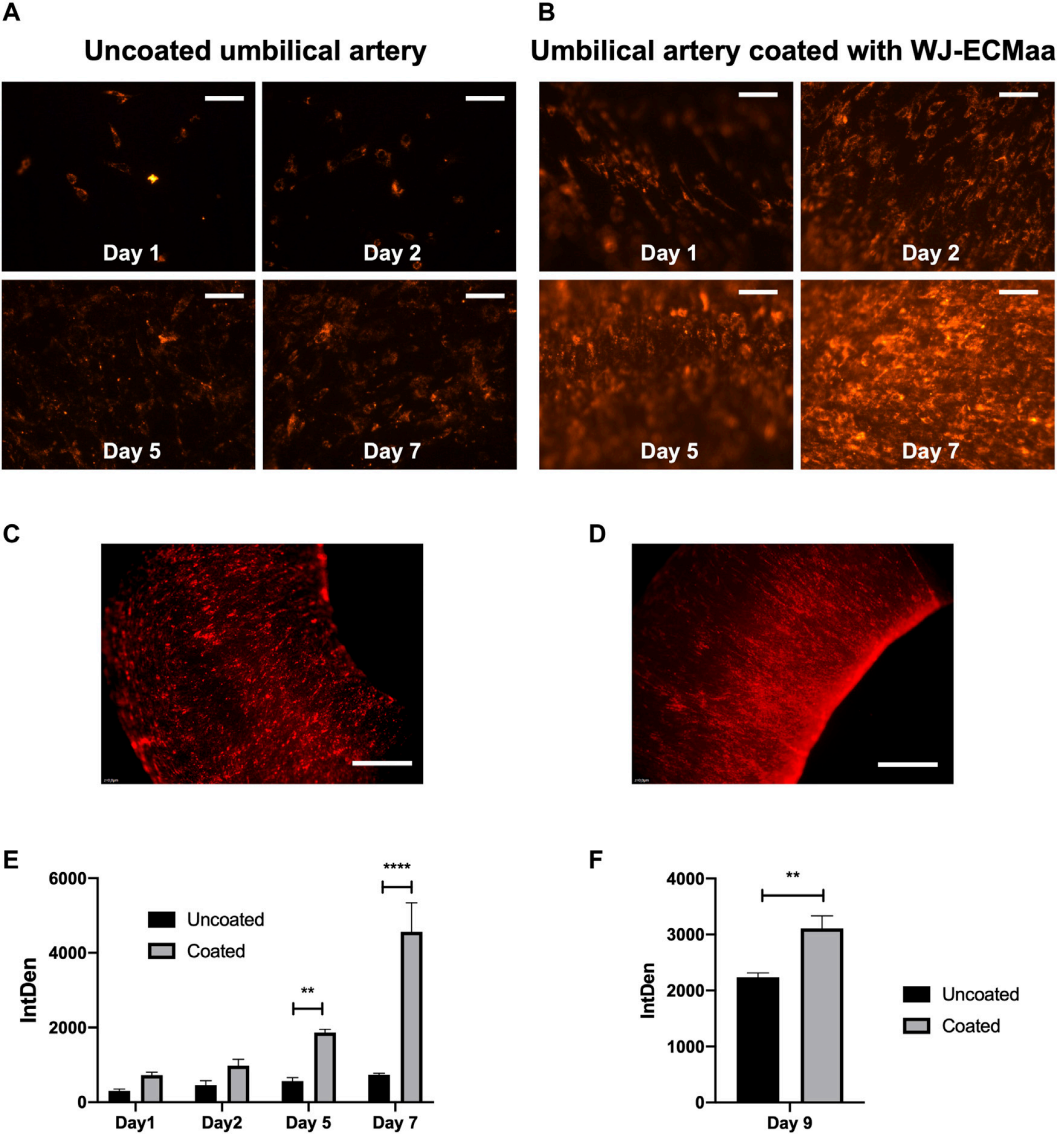
Evaluation of WJ-ECMaa coating impact on the cellularization process. Epifluorescence observations at days 1, 2, 5 and 7 of WJ-MSC labelled with DiI on uncoated **(A)** and previously coated **(B)** umbilical artery luminal surface (Scale bars, 500 µm). Epifluorescence Macroscopic observations at day 9 of both uncoated **(C)** and coated **(D)** segments (Scale bars, 1 mm). Comparison of integrated density at days 1, 2, 5, 7 **(E)** and day 9 **(F)** on uncoated and WJ-ECM coated segments. Results are presented as the mean ± SEM for each condition, and were statistically analyzed following two-way ANOVA (*p*-value: **< 0.01, ***< 0.0001); *n* = 3.

First day observations of the artery luminal surface indicate that, for two different segments of the same artery, and for an identical cell seeding density, a higher quantity of adherent WJ-MSC were found on the precoated segment (Figures 7A,B). Follow-up during the cell proliferation period showed a higher coverage of the luminal surface in the coated condition compared to the uncoated arterial segments. After 7 days of culture, the total coverage of the luminal surface is only observed for the WJ-ECMaa coated arterial segments (Figures 7A,B). All these results were supported by the integrated density analysis showing a higher value at each day for WJ-ECMaa coated segments in comparison to uncoated segments, with significant differences from day 5 (Figures 7E,F). At Day 9, macroscopic observations of larger areas of luminal surfaces of coated and uncoated segments have confirmed the previous results (Figures 7C,D).

## 4 Discussion

The ECM provides a microenvironment that plays a key role in promoting tissue remodeling, modulating gene expression and regulating cell functions such as adhesion, migration, proliferation and differentiation. Thus, it has had remarkable success when used as a scaffold in tissue engineering. Depending on the desired application, the initial conformation of the ECM may be retained, or ECM can be processed into other forms such as solutions that can be used as a coating applied to the surface of synthetic or biological biomaterials (Edgar et al., 2018).

Indeed, an ECM-derived coating will be of particular interest in the field of vascular tissue engineering where the development of functional and patent small-diameter tissue-engineered vascular grafts (TEVG) remains a considerable challenge. In fact, the failure of existing small-diameter vascular substitutes is due to various phenomena including thrombosis, intimal hyperplasia, infections and atherosclerosis. The occurrence of thrombosis, which is the major cause of vascular graft failure, can be explained by the absence of a functional endothelium on the luminal surface, which would prevent the adhesion of blood proteins and the triggering of coagulation mechanisms (Skovrind et al., 2019; Fayon et al., 2021). Therefore, having a uniform and continuous cell layer on the luminal surface of the graft seems to be a prerequisite for obtaining a hemocompatible small-diameter TEVG (Pashneh-Tala et al., 2016). Adding a luminal coating step prior to cellularization in the TEVG manufacture protocol is strongly recommended because it can create a microenvironment that can modify cell behavior by enhancing cell adhesion, proliferation and/or differentiation (Masson-Meyers and Tayebi, 2021). We are one of the multiple teams concerned by the lack of small-caliber vascular substitutes and seeking to develop a long-term patent and efficient small-diameter TEVG. In this study, we propose a luminal coating consisting of a natural complex ECM of human origin, extracted from the WJ of umbilical cords that is totally cytocompatible, reducing the cellularization period and suitable for various uses in tissue engineering.

Several methods of extracting ECM proteins have already been described in the literature, according to mechanical, chemical or enzymatic protocols, in order to obtain a product reflecting as much as possible the biological cell microenvironment (Moore et al., 2015; Schmidt, 2016; Coelho et al., 2017; Rahman, 2019).

We have previously described and characterized the WJ-ECM extracted by an enzymatic method (Dan et al., 2017a). In this study, we compared the results to those obtained after extraction of the same ECM in 0.02 N acetic acid that is commonly used to isolate collagen I (Rajan et al., 2006). This study has shown that the enzymatic extraction of WJ-ECM improves cell adhesion and proliferation in static and dynamic culture conditions, whereas extraction with a solution of 0.02 N acetic acid only allows the acquisition of very small quantities of matrix with unsatisfactory results. However, in order to produce a WJ-ECM under conditions that could be suitable for clinical applications, we decided to abandon the use of enzymes and to develop an efficient ECM extraction method with a higher concentration of acetic acid that can be more efficient than the one previously tested. This optimized extraction protocol allows the acquisition of ECM with an insignificant DNA content, thus making this biomaterial suitable for implantation protocols (Gilbert et al., 2009).

Proteomic analysis of the decellularized WJ has already been performed and determined that it is mainly composed of collagen, fibronectin and lumican (Jadalannagari et al., 2017). Except for fibronectin, these proteins are also found as the main components of the WJ-ECMaa, thus validating the interest that our method of preparation can carry. Analysis of the obtained deposit allowed us to highlight a particular arrangement of proteins, supposedly collagen fibers, in a fibril form. This arrangement of collagen fibers differs from the usually described conformation, a triple helix known to be the result of the collagen’s ability to self-assemble (Gross, 1964; Silver et al., 2003). In this case, the acetic acid extraction dismantles the collagen triple helix, allowing the extraction of collagen monomers (Davison et al., 1972). We assume here that the WJ collagen does not have the capacity to reform a triple helix, contrary to the collagen self-assembly described in the literature. Although collagen is a protein that has been intensively studied for a long time, many variations and subtypes of collagen are still being discovered (Ricard-Blum, 2011). In particular, collagen from fetal or neonatal tissue such as WJ seems to show some specificities (Vizza et al., 1996; Sobolewski et al., 1997). Despite advances in this field, it appears that the form of collagen found in our study has never been described before. It would be interesting to investigate and characterize the types of collagen found in neonatal tissue and to propose them as an alternatives for collagen type I of animal origin which is the most widely used for cell culture but also in tissue engineering applications (Cen et al., 2008; Parenteau-Bareil et al., 2010; Rico-Llanos et al., 2021).

Despite the unconventional structure of the collagen, it has allowed together with the other extracellular proteins found in the WJ-ECMaa, to obtain a uniform deposit with an optimal cytocompatibility. In fact, better cell adhesion, proliferation and metabolic activity were observed when WJ-MSC were seeded on WJ-ECMaa compared to the condition where they were seeded on a conventional collagen coating.

Hemocompatibility results have indicated that the WJ-ECMaa coating could have an interesting anticoagulant activity regarding its low values of ETP, peak and velocity index compared to the plasma (Tripodi, 2016). We suppose that this activity could be due to the presence of dermatan sulfate in the WJ, consistent with other studies (Gogiel et al., 2003; Yu et al., 2017; Sobczak et al., 2018), that have been demonstrated as a clotting regulator *via* the modulation of fibrin assembly (Dugan et al., 2006).

Endowed with a porous structure, an excellent cytocompatibility, and an anticoagulant action, the clinical tailored acetic acid-extracted ECM is perfectly suited as a covering surface for vascular substitutes since it would allow a more efficient cellularization of the luminal surface and prevent the coagulation phenomena that is a major cause of their failure.

As described above, the obtained solution allows to perform 2D surface coating resulting in a deposit of protein fibers according to the dip coating technique. It would be interesting to continue our characterization work in the case of dip coatings of porous materials, or according to different coating methods such as spray coating, which can be used for prosthesis covering (Monteiro et al., 2015).

The same method of dip coating could be used to coat the surface of a 3D scaffold or to impregnate a porous scaffold, whether synthetic or natural for tissue engineering applications. Indeed, as underlined by the presented proof of concept, using WJ-ECMaa to coat 3D biological tissues prior to cellularization, considerably improves the cell adhesion and proliferation and allows a full and homogeneous cell coverage.

The evaluation of obtained coating under different forms, as hydrogel by example, should be relevant and could allow its use in other contexts as a scaffold whose 3D structure could vary depending on the mold (Kopeček, 2007). Another improvement of such a coating could consist in the incorporation of functionalized nanoparticles allowing a controlled release of biomolecules.

## 5 Conclusion

In this study, WJ-ECMaa was analyzed to characterize this new biomaterial of human origin and recovered in a noninvasive manner. The decellularization and ECM isolation protocol is easy, short, noninvasive and non-expensive. To limit potential heterogeneity inherent to the natural and human-derived product, we chose to produce each batch of WJ-ECMaa by pooling three WJ derived from different umbilical cords. The three resulting batches of WJ-ECMaa, although presenting a relative quantitative heterogeneity for their protein, have shown homogeneous specifications. It has allowed to obtain a uniform porous biocompatible coating, showing a higher cytocompatibilty than commercially available collagen when seeded with WJ-MSC, cells of choice for tissue engineering. Moreover, it contains only a negligible amount of DNA, suggesting its potential use without the risk of an immune reaction in response to the insertion of foreign DNA. We were also able to perform proteomic analysis indicating its rich content in an unconventional form of human type I collagen. Although collagen is the main component of WJ-ECMaa, other proteins were also found including various proteoglycans known to promote cell adhesion and proliferation, in particular lumican (Wight et al., 1992; Nikitovic et al., 2008). Moreover, this coating has shown an interesting hypocoagulant potential that is a key feature for a use in the vascular engineering field.

In conclusion, all these characteristics make this natural ECM extracted from human umbilical cord a very promising biomaterial for cell culture and various tissue engineering applications, such as vascular tissue engineering due to its hypocoagulant potential. Its application as a vascular graft luminal coating can greatly improve the cellularization of this surface and avoids coagulation mechanisms.

## Data Availability

The original contributions presented in the study are included in the article/Supplementary Material, further inquiries can be directed to the corresponding author.

## Supplementary Material

Supplementary Figure S1 Chart pie of identified proteins composing the WJ-ECM.

Supplementary Figure S2 WJ-MSC phenotypic characterization by flow cytometry including 945 showing positive expression of CD90 (FITC anti-human CD90, BioLegend, 328108), CD44 946 (PE/Cy7 anti-human CD44, BioLegend, 338816), CD73 (Brilliant Violet 421TM anti-human 947 CD73, BioLegend, 344008), and CD105 (APC anti-human CD105, BioLegend, 323208) and a 948 lack expression of CD45 (PE anti-human CD45, BioLegend, 304008), CD34 (PE anti-human 949 CD34, BioLegend, 343506), HLA-DR (PE anti-human HLA-DR, BioLegend, 307606) and CD86 950 (PE anti-human CD86, BioLegend, 305406). The viable cells are identified by the use of the 951 Zombie AquaTM Fixable Viability Kit (BioLegend, 423101).
