# Supplementary figures and images for: Characterization of an Innovative Biomaterial Derived From Human Wharton’s Jelly as a New Promising Coating for Tissue Engineering Applications
Source: Front Bioeng Biotechnol. 2022 Jun 13;10:884069. doi: 10.3389/fbioe.2022.884069 (PMC9234273; doi:10.3389/fbioe.2022.884069)

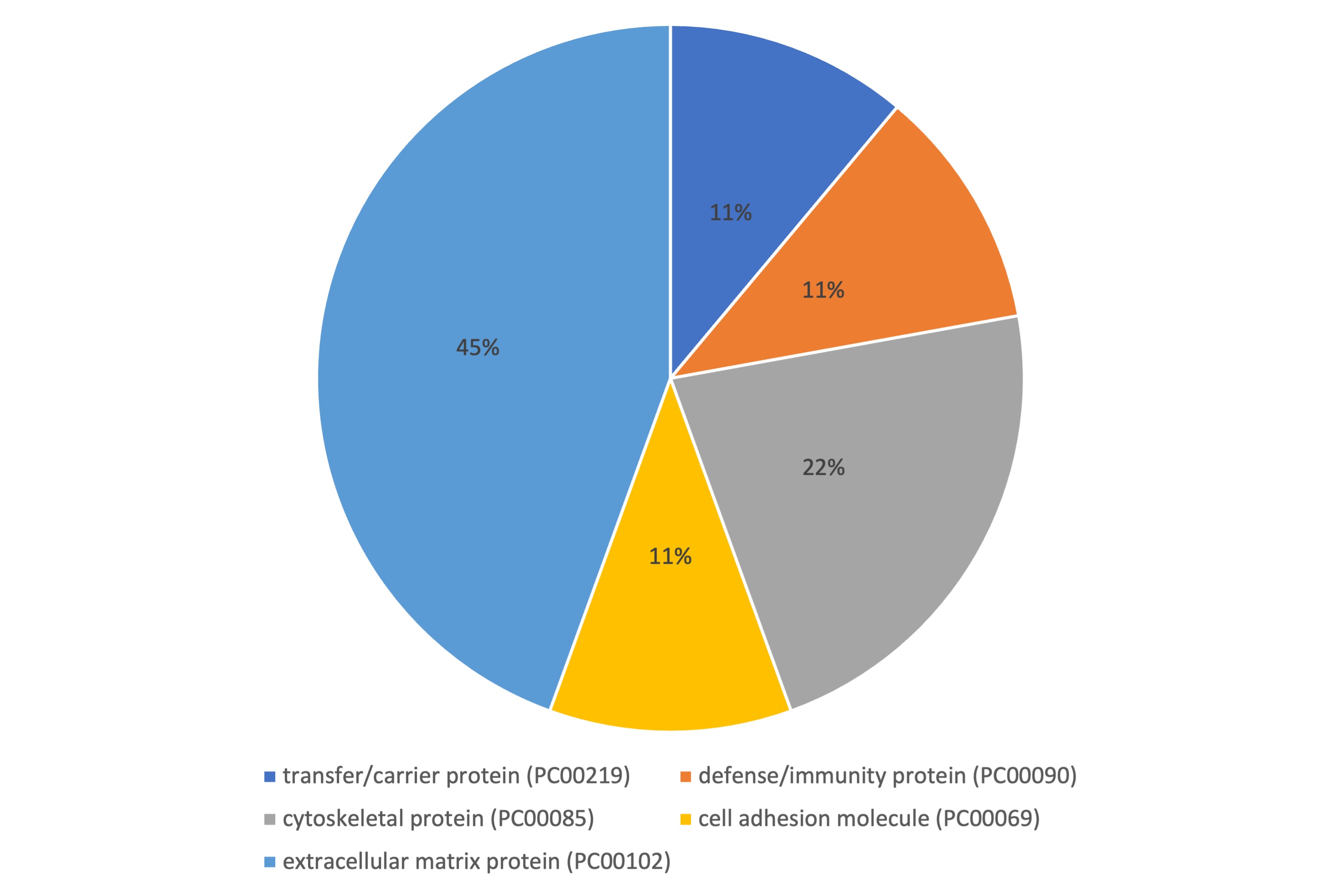

Supplement: Supplementary file 1 [file Image1.JPEG]

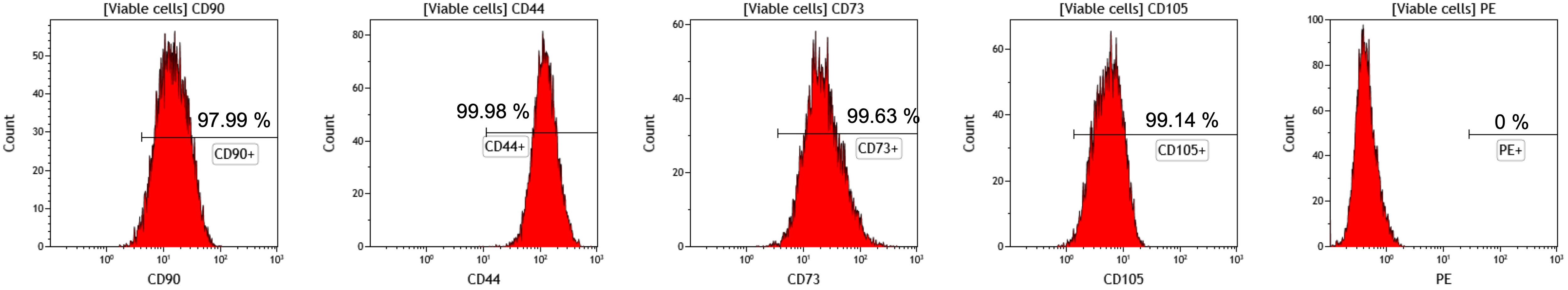

Supplement: Supplementary file 2 [file Image2.JPEG]
